# Expression of Concern: 24-Hour Rhythms of DNA Methylation and Their Relation with Rhythms of RNA Expression in the Human Dorsolateral Prefrontal Cortex

**DOI:** 10.1371/journal.pgen.1009470

**Published:** 2021-03-19

**Authors:** The PLOS Genetics Editors

Following publication of this article [1], concerns were raised regarding the methodology used for the reported analyses and whether the conclusions are fully supported.

The article included a reporting error pertaining to methods used for the analyses reported in Fig 2A,B of [1]. The Results and Materials and Methods sections incorrectly state, “we performed a series of analyses comparing the observed data to 10,000 permuted null datasets generated by randomly shuffling the times of death in our data while preserving the correlation between DNA methylation sites.” The authors have clarified that the analyses permuted time of death within each block of 500 contiguous CpG; the permutation is independent across the blocks, preserving local within block correlations. Thus, the Results and Materials and Methods statements should have read, "we performed a series of analyses comparing the observed data to 10,000 permuted null datasets generated by randomly shuffling the times of death in blocks of 500 contiguous CpGs in our data while preserving the within block correlation between DNA methylation sites.” The authors initially presumed that the block-wise permutation would yield identical results to an unblocked permutation. However, subsequent computations by the authors confirm that p-values obtained using this block-wise permutation approach are sensitive to block size.

Methodological concerns were raised pertaining to the use of a block-wise permutation approach, the use of averaged distributions for permuted null datasets, and use of shuffled times of death to compare distribution of nadir times. The authors acknowledge that when these aspects of the methodology are adjusted, some of the analyses reported in Figs 2 and 6 no longer yield statistically significant results.

The authors are cooperating with *PLOS Genetics* Editors to address these issues. In the meantime, the *PLOS Genetics* Editors issue this Expression of Concern.
